# Association of super-extended lymphadenectomy at radical cystectomy with perioperative complications and re-hospitalization

**DOI:** 10.1007/s00345-019-02769-9

**Published:** 2019-04-20

**Authors:** David D’Andrea, Mohammad Abufaraj, Francesco Soria, Kilian Gust, Andrea Haitel, Pierre I. Krakiewicz, Shahrokh F. Shariat

**Affiliations:** 1 Department of Urology, Medical University of Vienna, Währinger Gürtel 18-20, 1090 Vienna, Austria; 2 Division of Urology, Department of Special Surgery, Jordan University Hospital, The 2 University of Jordan, Amman, Jordan; 3 Division of Urology, Department of Surgical Sciences, AOU Città della Salute e della Scienza di Torino, Molinette Hospital, Turin, Italy; 4 Department of Pathology, Medical University of Vienna, Währinger Gürtel 18-20, Vienna, 1090 Austria; 5 Department of Urology, University of Montreal, Montreal, QC Canada; 6 Department of Urology, University of Texas Southwestern Medical Center, Dallas, TX USA; 7 Department of Urology, Weill Cornell Medical College, New York, NY USA; 8 Department of Urology, Second Faculty of Medicine, Charles University, Prag, Czech Republic; 9 Institute for Urology and Reproductive Health, I.M. Sechenov First Moscow State Medical University, Moscow, Russia

**Keywords:** Bladder cancer, Radical cystectomy, Lymphadenectomy, Complications

## Abstract

**Purpose:**

We performed a retrospective analysis of patients treated with radical cystectomy and lymphadenectomy (LAD) for bladder cancer to assess the differential association of the extent of LAD with perioperative complications and re-hospitalization.

**Materials and methods:**

LAD templates were defined as limited (lLAD = external, internal iliac and obturator), extended (eLAD = up to crossing of ureter and presacral lymph nodes), and super-extended (sLAD = up to the inferior mesenteric artery). Logistic regression models investigated the association of LAD templates with intraoperative, 30- and 30–90-day postoperative complications, as well as re-hospitalizations within 30 and 30–90 days.

**Results:**

A total of 284 patients were available for analysis. sLAD led to a higher lymph-node yield (median 39 vs 13 for lLAD and 31 for eLAD, *p* < 0.05) and N2/N3 status compared to lLAD and eLAD (*p* = 0.04). sLAD was associated with a blood loss of > 500 ml (OR 1.3, 95% CI 1.08–1.49, *p* = 0.003) but not with intraoperative transfusion, operation time, or length of hospital stay (*p* > 0.05). Overall, 11 (4%) patients were readmitted within 30 days and 50 (17.6%) within 30–90 days. The 30- and 30–90-day mortality rates were 2.8% and 1.4%, respectively. On logistic regression, LAD template was not associated with postoperative complications or re-hospitalization rates.

**Conclusions:**

sLAD leads to higher lymph-node yield and N2/N3 rate but not to higher complication rate compared to lLAD and eLAD. With the advent of novel adjuvant systemic therapies, precise nodal staging will have a crucial role in patients counseling and clinical decision making.

**Electronic supplementary material:**

The online version of this article (10.1007/s00345-019-02769-9) contains supplementary material, which is available to authorized users.

## Introduction

Radical cystectomy (RC) with lymphadenectomy (LAD), preceded by neoadjuvant chemotherapy (NAC) when possible, is the standard of care for muscle-invasive and recurrent high-risk bladder cancer (BCa) [1]. A properly done LAD allows accurate staging and may improve oncologic outcomes, since lymph-node metastases are present in up to 25% of patients treated with RC for muscle-invasive BCa [2–4]. However, the extent of LND is still under debate and the association of the extent of the LND templates with survival remains controversial [5]. In particular, the benefits of a super-extended LAD (sLAD) are questioned, as lymph-node metastasis above the aortic bifurcation is uncommon and the dissection of these nodes could result in longer operative times. Indeed, adverse events and increased complications can occur more frequently in more extensive LAD templates, potentially compromising quality of life in this population that is elderly in general [6–10]. The association of a more extensive LAD template with perioperative complications has not been sufficiently assessed, to our knowledge. Results from a recent randomized trial failed to show any significant differences in 30- and 90-day complications rates between extended LAD (eLAD) and sLAD [10]. However, this study did not report intraoperative complications and re-hospitalization rates. We hypothesized that a more extended LND would be associated with a higher rate of intra- and postoperative complications, length of stay, and re-admissions. To address this, we performed a detailed analysis of perioperative complications and investigated their association with different LAD templates.

## Materials and methods

### Patient selection

Following institutional review board approval (ID 2168/2017), we reviewed our institutional database to identify patients treated with RC and LAD for clinically non-metastatic BCa. Patients were considered non-metastatic if preoperative imaging did not show spread from the primary site to other places in the body. Patients with missing information regarding postoperative complications within 90 days and those who did not receive all oncologic care at our institution were excluded from the analysis.

### Intervention and pathological analysis

**Fig. 1:**
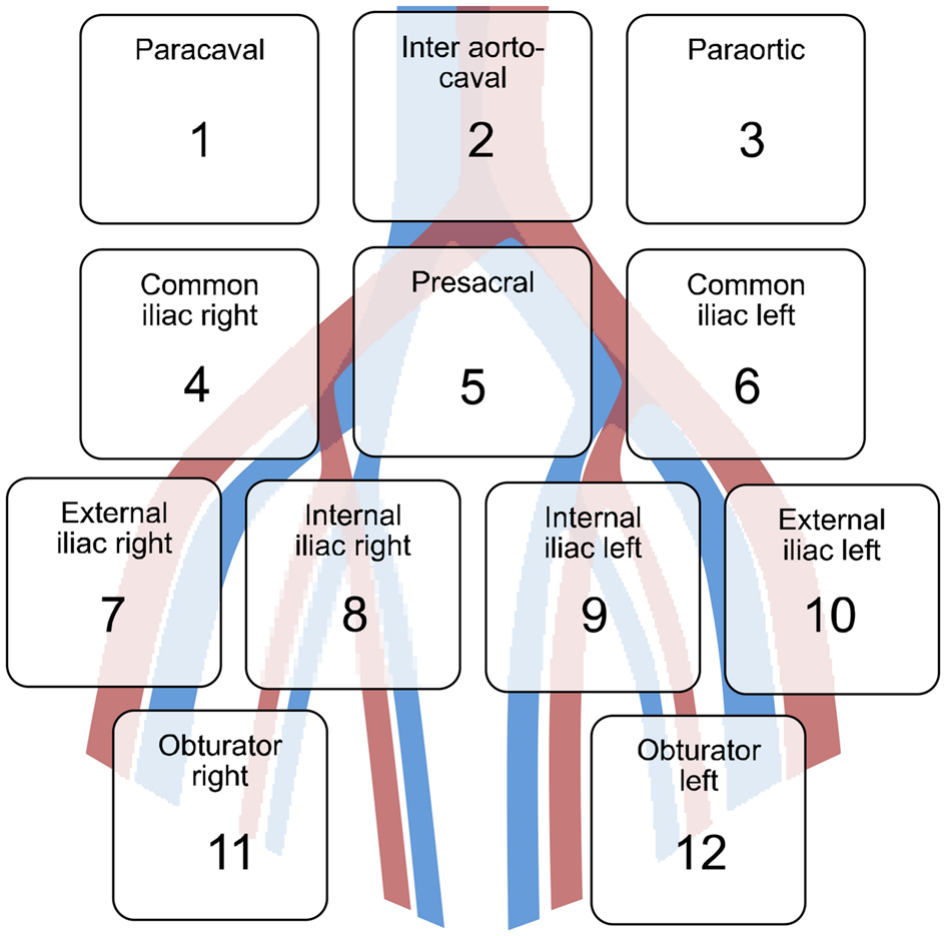
Anatomic template for lymph-node dissection. Limited template included internal iliac, external iliac, and obturator region (7–12). Extended template additionally included common iliac and presacral region (4–12). Super-extended template additionally included inferior mesenteric artery, paracaval, and para-aortal region (1–12)

Patients were treated with RC and LAD according to guideline recommendations at the time [1]. RC was performed by different surgeons. All procedures were performed by an open technique. The decision for the type of urinary diversions was based on disease characteristics, patient’s wishes, and performance status. The extent of LAD was at the surgeon’s discretion. We defined 12 LAD regions. The limited template (lLAD) included bilateral internal iliacs, external iliacs, and obturator regions; the extended template (eLAD) included lLAD plus up to the crossing of the ureter on the common iliac vessels and presacral region; and the super-extended template (sLAD) included the eLAD plus up to the inferior mesenteric artery in addition to, paracaval and para-aortal regions (Fig. 1). Specimens were analyzed by two dedicated genitourinary pathologists.

### Follow-up

Due to the retrospective nature of the study, the follow-up was not standardized, but was in accordance with the guidelines at the time. In general, patients underwent physical examination, ultrasonography, whole body imaging including CT with contrast or MRI as well as bone scan when indicated, and laboratory testing and at physician discretion based on patient and disease risk. Postoperative complications were assessed using the Clavien–Dindo classification [11]. Complications were recorded within 30 days after surgery and between 31 and 90 days after surgery. Major complications were defined as Clavien–Dindo ≥ 3. Cause of death was recorded based on chart and/or death certificate review [12].

### Statistical analyses

Means and standard deviations or median and interquartile range values are reported for normally or non-normally distributed continuous variables, respectively. Differences between proportions were reported using the Chi-square and the Mann–Whitney *U* test. We built logistic regression models to investigate the association of major postoperative complications as well as intraoperative complications with the LAD template used. Statistical significance was considered at a *p* < 0.05. All tests were two-sided. Statistical analyses were performed using R (R project, Vienna, Austria).

## Results

**Fig. 2:**
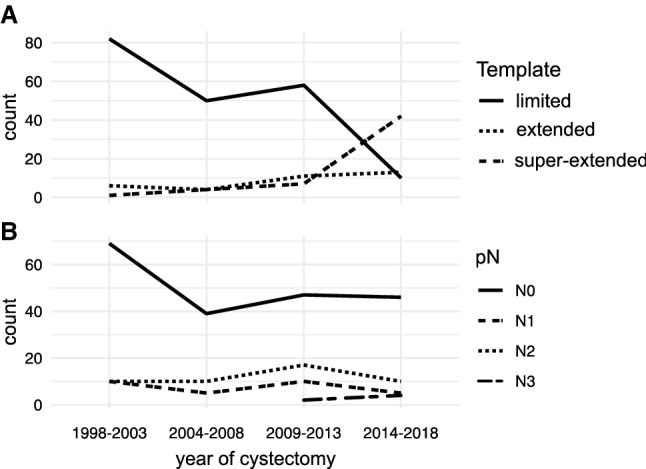
Distribution of lymphadenectomy templates (**a**) and pN status (**b**) over time in 284 patients treated with radical cystectomy for clinically non-metastatic bladder cancer between 1998 and 2018

We identified 434 patients treated with RC within our institutional database. A total of 284 patients had sufficient data available for analysis. Clinicopathologic features are depicted in Table 1. Overall, 200 (70.4%) were treated with lLAD, 34 (12%) with eLAD, and 50 with sLAD (17.6%). The use of sLAD increased with time (Fig. 2). This was associated with an increase in lymph nodes harvested (median nodes harvested 13 vs 31 vs 39 for lLAD, eLAD, and sLAD, respectively). sLAD was significantly associated with a higher incidence of N2 and N3 status at RC compared to lLAD and eLAD (*p* = 0.04). In subgroup analysis, the receipt of NAC in patients treated with sLAD was not associated with pN + status (OR 1.16, 95% CI 0.89–1.52).

**Table 1:** Clinicopathologic features and perioperative outcomes of 284 patients treated with radical cystectomy and lymphadenectomy (LAD) for clinically non-metastatic bladder cancer, stratified by LAD template

	Limited LAD	Extended LAD	Super-extended LAD	*p*
*n*	200	34	50	
Age, median (IQR)	67 (61–74)	65 (62–73.7)	68 (57.5–75.7)	0.92
Male gender (%)	158 (79.0)	25 (73.5)	37 (74.0)	0.63
BMI, median (IQR)	25.8 (22.9–27.96)	26.4 (23.9–29.7)	25.9 (22.4–27.96)	0.26
Charlson comorbidity index, median (IQR)	5 (4–7)	6 (4.2–8)	5 (4–8)	0.22
Preoperative hydronephrosis, *n* (%)				< 0.01
No	41 (20.5)	12 (35.3)	32 (64.0)	
Yes	13 (6.5)	9 (26.5)	17 (34.0)	
NA	146 (73.0)	13 (38.2)	1 (2.0)	
pT, *n* (%)				0.01
NMIBC	47 (23.5)	9 (26.5)	14 (28.0)	
T0	1 (0.5)	1 (2.9)	5 (10.0)	
T2	57 (28.5)	8 (23.5)	11 (22.0)	
T3	62 (31.0)	10 (29.4)	17 (34.0)	
T4	33 (16.5)	6 (17.6)	3 (6.0)	
pN, *n* (%)				0.04
N0	143 (71.5)	25 (73.5)	33 (66.0)	
N1	23 (11.5)	4 (11.8)	3 (6.0)	
N2	33 (16.5)	4 (11.8)	10 (20.0)	
N3	1 (0.5)	1 (2.9)	4 (8.0)	
Lymph nodes removed, median (IQR)	13 (7–22)	31 (13–37)	39 (25–52)	< 0.01
STSM, *n* (%)				0.28
R0	174 (87.0)	29 (85.3)	43 (86.0)	
R1	26 (13.0)	4 (11.8)	6 (12.0)	
Rx	0 (0.0)	1 (2.9)	1 (2.0)	
Variant histology, *n* (%)				< 0.01
Absent	175 (87.5)	22 (64.7)	32 (64.0)	
Mixed	15 (7.5)	11 (32.4)	12 (24.0)	
Pure	6 (3.0)	1 (2.9)	4 (8.0)	
NA	4 (2.0)	0 (0.0)	2 (4.0)	
Diversion, *n* (%)				0.03
UUCS	13 (6.5)	1 (2.9)	1 (2.0)	
Colon conduit	4 (2.0)	0 (0.0)	0 (0.0)	
Ileum conduit	127 (63.5)	30 (88.2)	43 (86.0)	
Ileum neobladder	54 (27.0)	3 (8.8)	5 (10.0)	
No diversion^a^	2 (1.0)	0 (0.0)	1 (2.0)	
NAC (%)	2 (1.0)	2 (5.9)	21 (42.0)	< 0.01
Days of hospital stay, median (IQR)	20 (16–25)	18 (15–20)	16.5 (13–25.7)	0.01
Transfusion rate, median (IQR)	1 (0–2)	0 (0–2)	1 (0–2)	0.71
Estimated blood loss (ml), median (IQR)	700 (400–1200)	700 (500–1225)	1000 (700–1200)	0.08
Operation time (min), median (IQR)	336 (281–402)	330 (295–360)	345 (295–370)	0.99

*IQR* interquartile range, *BMI* body mass index, *STSM* Soft tissue surgical margin, *UUCS* uretero–uretero-cutaneostomy, *NAC* neoadjuvant chemotherapy, *NA* not available

^a^No diversion necessary because of chronic hemodialysis

Regarding perioperative outcomes, sLAD was associated with a blood loss of > 500 ml compared to lLAD and eLAD (OR 1.3, 95% CI 1.08–1.49, *p* = 0.003). However, it did not affect the number of intraoperative transfusions, operation time, or length of stay (all *p* > 0.05). On multivariable analysis which adjusted for the effect of age-adjusted Charlson comorbidity index (CCI), tumor stage, body mass index (BMI), and the use of enhanced recovery after surgery (ERAS) protocol, sLAD remained associated with a blood loss of > 500 ml (OR 1.21, 95% CI 1.02–1.44, *p* = 0.03).

Overall, 30- and 30–90-day complication rates were 61.3% and 49.4%, respectively. Major complications occurred within 30 and 30–90 days in 75 (26.4%) and 41 (14.4%) patients, respectively. The 30- and 30–90-day mortality rates were 2.8% and 1.4%, respectively. A detailed distribution of postoperative complications is depicted in Tables 2 and s1. On logistic regression models, LAD template was not associated with postoperative major complications (*p* > 0.05) (Table 2). A total of 49 (17.2%) patients experienced a postoperative ileus within 30 days. On univariable logistic regression analyses, sLAE was associated with a lower risk of ileus (OR 0.89, 95% CI 0.79–0.99, *p* = 0.04). However, on multivariable analyses which adjusted for the effects of patient age, BMI, and the use of ERAS protocol, this association disappeared.

**Table 2:** Distribution of postoperative complications and logistic regression analyses investigating the association of the lymphadenectomy (LAD) template used with major postoperative complications (Clavien–Dindo ≥ 3) in 284 patients treated with radical cystectomy for clinically non-metastatic bladder cancer

	Complications within 30 days						Complications within 30–90 days					
	Clavien 0	Clavien 1–2	Clavien 3–4	Clavien 5	OR (95% CI)^a^	*p*	Clavien 0	Clavien 1–2	Clavien 3–4	Clavien 5	OR (95% CI)^a^	*p*
Limited LAD	78 (39%)	68 (34%)	48 (24%)	6 (3%)	Ref.		148 (74%)	25 (12.5%)	25 (12.5%)	2 (1%)	Ref.	
Extended LAD	15 (44%)	14 (41%)	5 (15%)	0	0.45 (0.15–1.14)	0.1	27 (79%)	4 (12%)	3 (9%)	0	0.62 (0.14–1.89)	0.4
Super-extended LAD	17 (34%)	17 (34%)	14 (28%)	2 (4%)	1.24 (0.62–2.39)	0.5	32 (64%)	6 (12%)	10 (20%)	1 (2%)	1.8 (0.8–3.87)	0.1

^a^Odds ratio reported for the occurrence of major complications

In all, 11 (4%) patients were readmitted within 30 days and 50 (17.6%) within 30–90 days after RC. On logistic regression models, unscheduled hospitalization rates within 30 and 30–90 days were not associated with the LAD template used (*p* > 0.05) (Table 3).

**Table 3:** Distribution and logistic regression analysis for the association of hospitalization rates with the lymphadenectomy (LAD) template used in 284 patients treated with radical cystectomy for clinically non-metastatic bladder cancer

	Hospitalizations within 30 days			Hospitalizations within 30–90 days		
	*N*	OR (95% CI)	*p*	*N*	OR (95% CI)	*p*
Limited LAD	6 (2.25%)	Ref.		34 (13.1%)	Ref.	
Extended LAD	3 (1.13%)	1.06 (0.99–1.45)	0.1	4 (1.5%)	0.95 (0.81–1.11)	0.5
Super-extended LAD	2 (0.75%)	1.01 (0.95–1.08)	0.7	13 (5%)	1.5 (0.71–3.19)	0.2

On exploratory analyses, we investigated the association of clinical features and performance scores with major complications and hospitalization. On logistic regression analysis, age (OR 1.03, 95% CI 1.01–1.06, *p* = 0.01) and CCI (OR 1.19, 95% CI 1.03–1.37, *p* = 0.01) were associated with 30-day major complications. Moreover, CCI (OR 1.24, 95% CI 1.05–1.47, *p* < 0.001) was associated also with 90-day major complications. BMI was associated with 30-day readmission (OR 1.14, 95% CI 1.01–1.27, *p* = 0.02) (Table 4).

**Table 4:** Logistic regression analyses investigating the association of clinical features with major postoperative complications (Clavien–Dindo ≥ 3) and hospitalization in 284 patients treated with radical cystectomy for clinically non-metastatic bladder cancer

	30-day major complications		30–90-day major complications	
	OR (95% CI)	*p*	OR (95% CI)	*p*
Age	1.03 (1.01–1.06)	0.01	1.03 (0.99–1.07)	0.1
Male gender	1.4 (0.74–2.79)	0.3	1.49 (0.66–3.82)	0.4
BMI	1.04 (0.78–1.1)	0.2	1.05 (0.97–1.23)	0.2
ASA	1.35 (0.6–2.99)	0.5	1.65 (0.66–4.1)	0.3
ECOG	1.21 (0.63–2.26)	0.5	1.46 (0.71–2.88)	0.3
CCI	1.19 (1.03–1.37)	0.01	1.24 (1.05–1.47)	< 0.01
ERAS	0.77 (0.21–2.23)	0.6	0.72 (0.11–2.69)	0.7
Diversion (no diversion or UUCS ref.)				
Conduit	1.42 (0.49–5.18)	0.5	3.52 (0.69–64.5)	0.2
Neobladder	0.93 (0.27–3.69	0.9	1.49 (0.22–29.6)	0.7

	30-day hospitalization		30-day hospitalization	
	OR (95% CI)	*p*	OR (95% CI)	*p*
Age	1.00 (0.95–1.06)	0.9	1.01 (0.98–1.04)	0.5
Male gender	1.32 (0.33–8.82)	0.7	1.39 (0.66–3.23)	0.4
BMI	1.14 (1.01–1.27)	0.02	1.03 (0.96–1.1)	0.3
ASA	0.83 (0.08–6.14)	0.9	0.8 (0.3–1.99)	0.6
ECOG	2.39 (0.56–8.68)	0.2	1.08 (0.49–2.15)	0.8
CCI	1.13 (0.81–1.49)	0.4	1.13 (0.95–1.32)	0.1
ERAS	1.5 (0.08–8.58)	0.7	1.37 (0.37–4.01)	0.6
Diversion (no diversion or UUCS ref.)				
Conduit	0.34 (0.05–6.85)	0.3	3.89 (0.76–71.2)	0.2
Neobladder	1.82 (0.28–35.7)	0.6	3.67 (0.64–69.5)	0.2

*OR* odds ratio, *CI* confidence interval, *BMI* body mass index, *ASA* American Society of Anesthesiologists, *ECOG* Eastern Cooperative Oncology Group, *CCI* Charlson comorbidity index; *ERAS* enhanced recovery after surgery

## Discussion

We investigated perioperative complications and hospitalization rates associated with lLAD, eLAD, and sLAD using data originating from a tertiary referral center. We found that two-thirds of patients experienced any type of complication and one-third had at least one major complication within 30 days after RC. Our results corroborate those reported in retrospective studies and prospective trials [3, 5, 9, 10, 13–15]. To our knowledge, this is the first study investigating the association of lLAD, eLAD, and sLAD with perioperative outcomes. We reject our null hypothesis, as we failed to observe an association between the extent of LAD template and perioperative complications. Importantly, the previous reports did not investigate the impact of LAD template on surgical morbidity. A retrospective multicenter series investigated the association of LAD with survival outcomes. Authors could not show a significant difference between eLAD and sLAD. However, no postoperative outcomes were reported [9]. Results from the first RCT comparing lLAD vs sLAD did not show significant difference in terms of recurrence-free survival between groups. On exploratory analyses, mortality and major complications (Clavien–Dindo ≥ 3) occurring within 30 and 90 days did not differ between groups [10].

We found that re-hospitalization rates were lower than those reported in large series (19.6% vs > 40%) [16, 17]. However, the comparative data originate from population-based databases including different hospitals. Indeed, data from high-volume tertiary care centers revealed similar re-hospitalization rates compared to ours [3, 18].

While clinicopathologic features and comorbidity indices have been associated with hospitalization [3, 18], studies investigating this association with LAD templates are missing. We filled this gap using logistic regression models.

We did not find any difference in operative times between different LAD templates. Retrospective series reported controversial results [13, 19]. The operative time depends on the surgeon, different LAD templates, previous surgeries, and other factors affecting the surgical site; all of these may have influenced these results. We have adopted sLAD in more recent years, reflecting the improvement of the surgical technique and a change in surgical paradigm over the years. Although the sLAD cohort had higher blood loss, this was not associated with higher transfusion rates [13]. Various factors can affect blood loss such as comorbidities, surgical approach and technique, disease severity as well as measurement of blood loss. To our knowledge, no previous studies have reported on blood loss in patients who underwent sLND.

LAD is an essential compound of RC necessary for accurate staging and, potentially, for disease control [5]. A recent RCT form Germany highlighted this issue reporting a 2% rate of lymph-node metastases outside the lLAD [10]. In our series, four patients (8%) treated with sLAD had lymph-node metastases outside the lLAD and eLAD template. These patients would have been, therefore, understaged if no sLAD would have been performed. Moreover, we found higher rates of pN3 compared to this trial (2.1% vs 0.5%). However, these results must be interpreted in the context of the retrospective design and the lack of pathologic re-review. It is, however, true that our series includes patients with clinically more evolved disease than the German RCT.

Identification of preoperative risk factors for hospitalization is of paramount importance for patients’ management and counseling. In line with the previous reports, we identified advanced age and high CCI to be associated with the risk of major postoperative complications [13, 14, 19]. However, these studies did not investigate the association of LAD extent with outcomes. This is a relevant shortcoming, as omitting surgical steps can obviously influence perioperative outcomes. To overcome these limitations, we performed a detailed analysis of complications associated with different LADs and showed that a more extensive LAD is not associated with higher perioperative morbidity, while this is associated with a better disease staging. Whether the extent of LAD is associated with cure rates and survival remains to be assessed in ongoing prospective RCTs (i.e., SWOG 1011).

Nevertheless, RC remains a procedure associated with high perioperative complication rates due to the procedure itself, patients’ comorbidities and tumor characteristics. It is, therefore, of paramount importance to balance potential benefits of a more extended LAD and counsel patients for a risk-adapted approach. In this context, one-fits-all approach using a unified template for all patients is not optimal and can be considered an overtreatment in a significant number of patients [6, 7]. There is an unmet need for better patient selection and development of clinical tool which may aid in the identification of patients who are more likely to benefit from a sLAD.

Despite its strengths, our study is not devoid of limitations, which are mainly inherent to its retrospective design. The long time span of the study and missing sLAD in early years may have influenced results. Moreover, different surgeons over time may have also introduced additional bias. sLAD patients had higher rate of NAC which has been suggestive to lead to more difficult dissection and a minimally higher complication rate [20]. Finally, other perioperative pathways that have shown to improve surgical outcomes, i.e., the ERAS protocol [21], were implemented in more recent patients treated mainly with sLAD, influencing, therefore, results.

## Conclusions

Our data confirm that while sLAD leads to higher number of nodes removed and resulting higher nodal stage, it does not increase perioperative morbidity. Further studies should focus on the development of risk-adapted models to guide the choice of an individualized LAD template based on clinicopathologic characteristics and patient health status in a shared decision-making process with the patient.

## Electronic supplementary material

Below is the link to the electronic supplementary material.

Supplementary material 1 (DOCX 12 kb)
