# Supplementary material for: Association of super-extended lymphadenectomy at radical cystectomy with perioperative complications and re-hospitalization
Source: World J Urol. 2019 Apr 20;38(1):121–8. doi: 10.1007/s00345-019-02769-9 (PMC6954123; doi:10.1007/s00345-019-02769-9)
Supplement: Supplementary file 1 — Supplementary material 1 (DOCX 12 kb) [file 345_2019_2769_MOESM1_ESM.docx]

**Table s1 - Distribution of postoperative complications in 284 patients treated with radical cystectomy and lymphadenectomy (LAD) for clinically non-metastatic bladder cancer, stratified by LAD template.**

|  | **Limited LAD** | **Extended LAD** | **Super-extended LAD** |
| --- | --- | --- | --- |
| **Complication within 30-days, n (%)** | | | |
| Ileus | 39 (19.5) | 6 (17.6) | 4 (8) |
| Gastrointestinal | 18 (9) | 3 (8.8) | 5 (10) |
| Infection | 36 (18) | 8 (23.5) | 11 (22) |
| Wound healing | 31 (15.5) | 2 (5.9) | 14 (28) |
| Cardio-pulmonal* | 36 (18) | 8 (23.5) | 18 (36) |
| Metabolic | 6 (3) | 2 (5.9) | 1 (2) |
| Lymphocele | 7 (3.5) | 0 | 2 (4) |
| Neurological | 11 (5.5) | 1 (2.9) | 4 (8) |
| Genitourinary** | 12 (6) | 1 (2.9) | 6 (12) |
| **Complication within 30-90-days, n (%)** | | | |
| Ileus | 3 (1.5) | 1 (2.9) | 0 |
| Gastrointestinal | 11 (5.5) | 0 | 1 (2) |
| Infection | 30 (15) | 4 (11.7) | 12 (24) |
| Wound healing | 10 (5) | 1 (2.9) | 1 (2) |
| Cardio-pulmonal* | 8 (4) | 1 (2.9) | 1 (2) |
| Metabolic | 11 (5.5) | 1 (2.9) | 2 (4) |
| Lymphocele | 4 (2) | 0 | 2 (4) |
| Neurological | 1 (0.5) | 0 | 1 (2) |
| Genitourinary** | 7 (3.5) | 0 | 4 (8) |

* Cardio-pulmonal includes also thrombosis, embolism and transfusions

**includes ureteral stent dislocation requiring reposition, nephrostomy position and anastomosis/neobladder leak

Multiple complications per patient can occur
